# Regulation of Metabolic Reprogramming by Long Non-Coding RNAs in Cancer

**DOI:** 10.3390/cancers13143485

**Published:** 2021-07-12

**Authors:** Assunta Sellitto, Giovanni Pecoraro, Giorgio Giurato, Giovanni Nassa, Francesca Rizzo, Pasquale Saggese, Cesar A. Martinez, Claudio Scafoglio, Roberta Tarallo

**Affiliations:** 1 Laboratory of Molecular Medicine and Genomics, Department of Medicine, Surgery and Dentistry ‘Scuola Medica Salernitana’, University of Salerno, 84081 Baronissi, Italy; assellitto@unisa.it (A.S.); gipecoraro@unisa.it (G.P.); ggiurato@unisa.it (G.G.); gnassa@unisa.it (G.N.); frizzo@unisa.it (F.R.); 2 Genome Research Center for Health—CRGS, University of Salerno Campus of Medicine, 84081 Baronissi, Italy; 3 Division of Pulmonary and Critical Care Medicine, David Geffen School of Medicine, University of California Los Angeles, Los Angeles, CA 90095, USA; psaggese@mednet.ucla.edu (P.S.); cesaramartinez@mednet.ucla.edu (C.A.M.); cscafoglio@mednet.ucla.edu (C.S.)

**Keywords:** cancer metabolism, long non-coding RNAs, metabolic reprogramming, glycolysis, Warburg effect, glutaminolysis, lipid metabolism

## Abstract

**Simple Summary:**

The molecular interplay between long non-coding RNAs (lncRNAs) and cancer metabolic reprogramming enables malignant cells to adjust metabolic reactions and nutrient uptake, supporting tumor growth and dissemination. Here, we summarize the current background on lncRNA-driven alterations of cell metabolic processes, with a particular emphasis on hypoxia-inducible pathways, glycolytic process, oxidative phosphorylation, lipid anabolic and catabolic reactions, amino acid metabolism and signal transduction pathways, with the main aim of elucidating the complex network of interactions between metabolism and lncRNA expression and activity. Indeed, due to their pleiotropic roles in cell physiology and cancer development and progression, lncRNAs are currently guarded as promising diagnostic and prognostic biomarkers and therapeutic targets, providing a novel approach for the early diagnosis and personalized therapy of multiple neoplasms.

**Abstract:**

Metabolic reprogramming is a well described hallmark of cancer. Oncogenic stimuli and the microenvironment shape the metabolic phenotype of cancer cells, causing pathological modifications of carbohydrate, amino acid and lipid metabolism that support the uncontrolled growth and proliferation of cancer cells. Conversely, metabolic alterations in cancer can drive changes in genetic programs affecting cell proliferation and differentiation. In recent years, the role of non-coding RNAs in metabolic reprogramming in cancer has been extensively studied. Here, we review this topic, with a focus on glucose, glutamine, and lipid metabolism and point to some evidence that metabolic alterations occurring in cancer can drive changes in non-coding RNA expression, thus adding an additional level of complexity in the relationship between metabolism and genetic programs in cancer cells.

## 1. Introduction

In the last decades, advances in the Next-Generation Sequencing (NGS) technologies fueled the discovery of several classes of non-coding RNAs (ncRNAs), generating a great interest in the scientific community for their pleiotropic functions in the regulation of gene expression in physiological processes as well as in pathologic conditions, where their dysregulation strongly contributes to genetic and epigenetic aberrations [1]. According to their length, ncRNA molecules can be roughly classified into small non-coding RNAs (sncRNAs) of 18–35 nt, such as miRNAs, piRNAs and siRNAs, and long non-coding RNA transcripts (lncRNAs) ranging from 200 nt to 100 kilobases.

The different ncRNA classes participate in multiple interconnected pathways, controlling chromatin architecture and transcription in the cell nucleus and modulating the expression of target genes in the cytoplasm by influencing mRNA stability, translation and post-translational modifications [2].

miRNAs, one of the best-characterized classes of sncRNAs, regulate a wide range of biological processes, including cell proliferation, development and cell-fate specification, while piRNAs, in complex with evolutionally conserved PIWI proteins, have been associated with the regulation of transposon activity and genome protection. The long non-coding RNA (lncRNA) class includes long intergenic non-coding RNAs (lincRNAs), natural antisense transcripts (NATs), transcribed ultraconserved region (T-UCR) and non-coding pseudogenes. lncRNAs regulate stem-cell maintenance and cell differentiation, embryogenesis, X-chromosome inactivation, imprinting and the establishment of epigenetic marks [3,4,5,6,7].

Based on their molecular function, lncRNAs can regulate the transcription process, by interacting with transcription factors (TFs) and other proteins at transcription start sites on the chromatin, but they can also have structural roles, acting as “molecular scaffolds” for protein complexes and taking part in large tridimensional structures with regulatory functions, such as ribonucleoprotein and chromatin-modifying complexes, where lncRNAs adopt complex structures by interacting with DNA, RNA and proteins. In addition, lncRNAs can also regulate the activity of other ncRNAs such as miRNAs, acting as ceRNAs (competing endogenous RNAs) and sequestering them by directly interacting with miRNA-binding regions, thereby regulating target mRNA expression [8,9,10].

In recent years, ncRNAs have emerged as key regulators of the major hallmarks of cancer, including metabolic reprogramming, that enables malignant cells to adjust metabolic reactions and nutrient uptake to support their accelerated proliferation, tumor growth and dissemination [11,12]. Increasing evidence demonstrates that sncRNAs are often deregulated in cancer cells and can be drivers of cancer transformation; “oncomiRs” and “tumor suppressor miRNAs” have been described, whose activity leads to the activation of oncogenic pathways through the expression of oncogenes or the inactivation of tumor suppressor genes [13]. Furthermore, several lncRNAs with critical roles in cancer development have been characterized, such as the Metastasis Associated Lung Adenocarcinoma Transcript 1 (MALAT1), promoting tumor growth and invasion [14]. Another example is the HOX Transcript Antisense Intergenic RNA (HOTAIR), highly expressed in several cancers, which recruits histone-modifying complexes to target genes establishing suppressive histone marks [15]. Other lncRNAs such as Prostate Cancer Gene Expression Marker 1 (PCGEM1) are involved in the regulation of metabolic genes acting as coactivators of the androgen receptor [16].

By reviewing the state of the art concerning the involvement of lncRNAs in cancer cell metabolism, we aimed at elucidating the molecular circuits regulated by these ncRNAs that could be of interest in the clinical practice for their involvement in the mechanisms of carcinogenesis and cancer progression. The development of new lncRNA-based diagnostic/prognostic tools and gene-editing therapies is currently emerging as a promising perspective in the field of targeted therapy and personalized medicine.

## 2. Metabolic Regulation of lncRNAs

**Figure 1:**
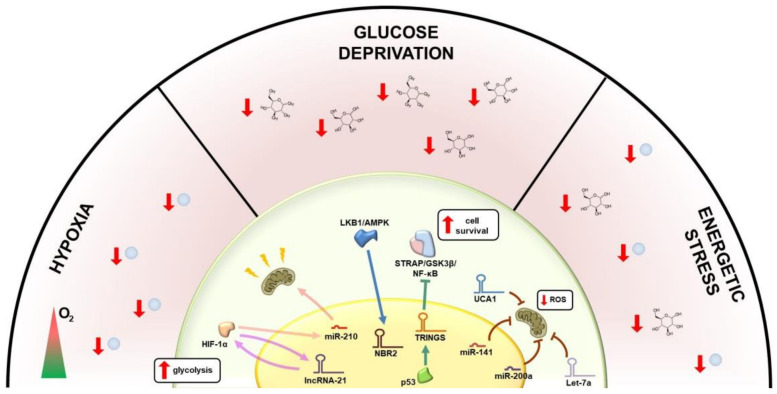
Non-coding RNAs promote the ability of cancer cells to survive in a hostile microenvironment. Low-oxygen conditions, glucose deprivation and energetic stress are major inductors of perturbation in non-coding RNA expression and interaction with cellular metabolism. Under hypoxic conditions, the cross-talk between the transcription factor HIF-1α (hypoxia-inducible factor 1-alpha) lncRNA-21and miR-210 suppresses mitochondrial function and promotes glycolysis. Glucose starvation up-regulates the lncRNA TRINGS (Tp53-regulated inhibitor of necrosis under glucose starvation), thus inhibiting the STRAP–GSK3β–NF-κB necrotic signaling: the liver kinase B1 (LKB1)/AMP-activated protein kinase (AMPK regulates the expression of the lncRNA NBR2 (neighbor of BRCA1 gene 2), resulting in cell cycle progression. miR-141, miR-200a, Let-7a and UCA1 participate in the fine-tuning of the oxidative phosphorylation in mitochondria thus modulating the oxidative stress.

Within the tumor microenvironment, the metabolic stress experienced by cancer cells is a major inductor of perturbation in ncRNA expression: metabolic signals regulate ncRNA activity, which in turn modulates the expression of metabolic enzymes and signaling pathways implicated in glucose, lipid and amino acid metabolism, as well as mitochondrial respiration (Figure 1) [11,12,17,18].

Metabolic signals can regulate ncRNA expression and functions; for example, hypoxia-regulated miRNAs have been described, some of which up-regulated while others down-regulated within the tumoral hypoxic environment [19]. The crosstalk between lncRNAs and cellular metabolism also has important implications in cancer progression; for example, the lncRNA-21 is positively regulated by c-Myc and HIF-1α, two transcription factors with a key role in glucose metabolism; lncRNA-21, in turn, binds to HIF-1α preventing its degradation and leading to hypoxia-enhanced glycolysis [20] (Figure 1). Energy stress is responsible for changes in lncRNA expression; for example, the lncRNA NBR2 (neighbor of BRCA1 gene 2) is induced by the liver kinase B1 (LKB1)/AMP-activated protein kinase (AMPK), a critical sensor of the cellular energy status; interestingly, the knockdown of NBR2 results in uncontrolled cell cycle progression and enhanced tumor growth (Figure 1); in agreement with this, low NBR2 expression is an indicator of poor prognosis in several human cancers [16].

Cancer cells reprogram their metabolism based on complex regulatory networks involving ncRNAs and their molecular target, including key-enzymes and factors with oncogenic or tumor suppressor activities. Among them, the tumor suppressor p53, a well-known transcription factor that regulates many stress response genes including ncRNAs, plays a critical role. p53 is activated in normal cells under stress conditions to prevent malignant transformation; however, cancer cells also need to survive in a hostile tumor microenvironment with low nutrients and oxygen. A recent study has pointed out a role for p53 in aiding tumor cell survival [21]. Indeed, under glucose starvation, wild-type p53 transcriptionally up-regulates the lncRNA TRINGS (Tp53-regulated inhibitor of necrosis under glucose starvation), in cancer cells, promoting their survival [21]. On the other hand, TRINGS binds to STRAP and inhibits the STRAP–GSK3β–NF-κB necrotic signaling protecting tumor cells from death [21] (Figure 1). Another lncRNA, GLCC1, is significantly up-regulated upon glucose starvation in CRC cells; GLCC1 interacts with HSP90 chaperon and regulates c-Myc stability by impairing its degradation via ubiquitination. As a consequence, c-Myc target genes, such as LDHA, are positively regulated, enhancing the glycolysis and supporting cell survival and proliferation [22].

Cancer cell dependence on glycolysis for energy production is also promoted by the hypoxic microenvironment. For example, miR-210, up-regulated by hypoxia, suppresses mitochondrial function through down-regulation of the electron transport chain complexes and iron homeostasis in colon, breast and esophageal cancers [23]. Mitochondrial reprogramming is essential to avoid the production of reactive oxygen species (ROS), a by-product of the electron transport chain, and is regulated by nuclear and mitochondrial ncRNAs. The control of tumorigenic signals modulating ROS levels can be mediated by ncRNAs (Figure 1) [24]. For example, in lung cancer miR-17-92 [25] reduces DNA damage, leading to the accumulation of genetic instability, while in ovarian cancer, miR-141 and miR-200a modulate the oxidative stress by targeting p38α [26]. Let-7a and urothelial carcinoma-associated 1 (UCA1) lncRNAs also participate in ROS formation (Figure 1), with the former targeting PDK1 in breast [27] and hepatocellular carcinoma [28], and the latter promoting oxidative phosphorylation and mitochondrial glutaminolysis, acting as a sponge for miR-16 in bladder [29]. Interestingly, mitochondrial DNA-encoded lncRNAs and miRNAs (mitomiRs) with either pro- or anti-oxidant activities have been described, most of them participating in the fine-tuning of oxidative phosphorylation [11].

## 3. Regulation of Glucose Metabolism by lncRNAs

**Figure 2:**
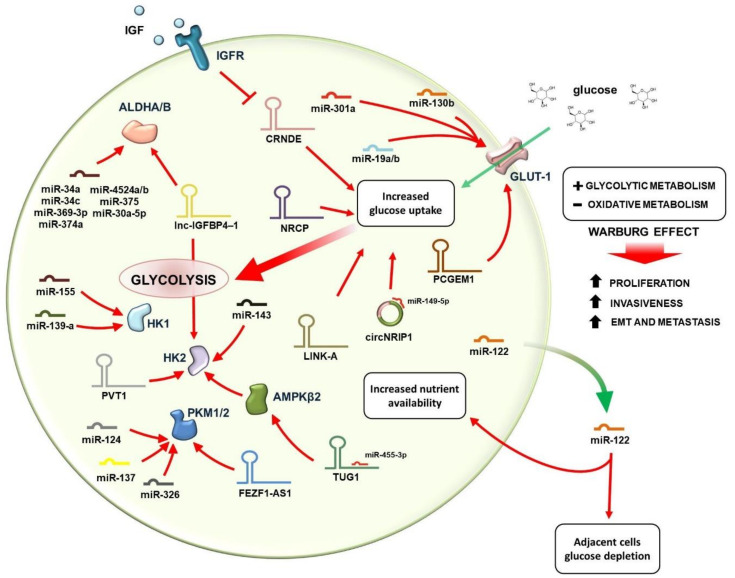
The Warburg effect drives cancer cells energetic independence, survival and ability to disseminate. Cancer cells promote glucose supply by increasing its uptake; glucose influx is regulated by miR-130b, miR-19a/b, miR-301 and PCGEM1 by inducing the overexpression of glucose transporters such as GLUT-1, or by subtracting it from normal cells within the tumor microenvironment through the secretion of miRNAs such as miR-122. Glucose uptake can be also mediated by circular RNAs, such as circNRIP1, sponging miR-149-5p and other lncRNA such as NRCP, LINK-A and by the CRNDE (Colorectal Neoplasia Differentially Expressed) lncRNA via insulin-like growth factor (IGF) stimulation. Hexokinase enzymes (HK1, HK2) control the rate of glucose metabolism and are up-regulated by complicated networks including several miRNAs, lncRNAs and other glycolytic enzymes such as AMPKβ2 (adenosine monophosphate-activated protein kinase subunit β2) and the pyruvate kinases PKM1 and PKM2. Red arrows indicate a positive regulation, while truncated red arrows indicate inhibitory mechanisms.

One of the main characteristics of tumor cells is an accelerated energetic demand to support their rapid proliferation and biosynthetic reactions; the main nutrient used by cancer cells to produce energy for tumor growth is glucose, whose carbon bonds are oxidized to generate ATP [17]. Cancer cells increase their energetic metabolism by adopting two main strategies: first, malignant cells promote glucose supply by increasing its uptake and by subtracting it from normal cells within the tumor microenvironment; second, they prevent energy production through mitochondrial oxidative phosphorylation, generally preferred in normal cells, while accelerating the glycolysis pathway, less efficient in ATP production but more rapid [12]. As a consequence of this phenomenon, known as the Warburg effect, even in the presence of oxygen and fully functioning mitochondria, cancer cells become energetically independent and resistant to anoikis death, increasing their probability of survival and acquiring the ability to metastasize [30]. Cancer cells reprogram their glucose metabolism based on a complex regulatory network, made of molecular circuits involving several oncogenes and tumor-suppressor genes, whose expression is often modulated by ncRNAs (Figure 2).

One mechanism by which cancer cells promote glucose uptake is the increase in exposition of glucose transporters (GLUTs), transmembrane glycoproteins distributed on the cell membrane, whose expression is regulated by ncRNAs in several malignancies [11]. *GLUT-1* over-expression and consequent glucose influx, for example, are regulated by miR-130b, miR-19a/b and miR-301a in renal clear cell tumors, and by the lncRNA PCGEM1 in prostate cancer (Figure 2), where the expression of the gene is also associated with invasiveness and matrix metalloproteinase-2 (MMP-2) activity [31]. Glucose uptake regulation by lncRNAs is fairly common in cancer. In colorectal cancer, indeed, the Colorectal Neoplasia Differentially Expressed (CRNDE) lncRNA promotes glucose uptake upon insulin-like growth factor (IGF) stimulation (Figure 2) [32]. In ovarian and breast cancer cells, overexpression of the ceruloplasmin lncRNA (NRCP) promotes glucose uptake and enhances tumor progression (Figure 2); a similar effect has been observed in triple-negative breast cancer, where glucose uptake is modulated by the LINK-A lncRNA [11,33]. In addition, in metastatic breast cancer, high levels of miR-122 secreted by the tumor repress glucose uptake in normal cells (Figure 2), thus increasing the nutrient availability for cancer cells [11,34]. In gastric cancer, interestingly, glucose uptake and ATP production are also controlled by the circular RNA circNRIP1, which binds to miR-149-5p activating the AKT/mTOR signaling pathway, regulating glucose utilization and promoting metastasis (Figure 2) [35].

After glucose entry into the cell, several ncRNA-modulated glycolytic enzymes take part in its catabolism. Hexokinases (HKs), the first enzymes of the glycolytic pathway, control the rate of glucose metabolism and are frequently up-regulated in cancer, thus maintaining a high glycolytic rate. Oncogenic miRNAs influence the activation of HK enzymes, which in turn exert a pro-tumorigenic activity and facilitate dissemination (Figure 2). HK1 is regulated in cancer cells by miR-155 [11,36] and miR-139-5p [12,37]; HK2 is overexpressed in a variety of malignancies including head and neck, lung, colon, cervical, bladder and prostate cancers through the down-regulation of miR-143 [11,38]. In ovarian cancer, HK2 enhances the expression of MMP-9, SOX-9 transcription factor and NANOG pseudogene, facilitating metastasis process [12,39]. Long non-coding RNAs participate in this complicated network by modulating the activation of glycolytic enzymes, a function that can be also performed by affecting the expression of some miRNAs. In hepatocellular carcinoma (HCC), the lncRNA TUG1 regulates the expression of miR-455-3p, which targets the adenosine monophosphate-activated protein kinase subunit β2 (AMPKβ2), involved in the regulation of HK2; TUG1 is strongly associated with HK2 overexpression, enhanced glycolysis and cell migration, and represents an indicator of poor prognosis in HCC [40]. In gallbladder cancer, the lncRNA PVT1 is frequently up-regulated and associated with glucose metabolism via HK2 modulation [11,12,41]. Another key enzyme of the pathway is aldolase, which is targeted by miR-122 in the liver [11,42]. In liver cancer, in addition, the up-regulation of hypoxia factors suppresses the expression of miR-199a-5p and promotes glycolysis, while in colorectal cancer, miR-155 promotes the Warburg effect via the IL-6/STAT3 pathway [36].

The terminal enzyme of glycolysis, pyruvate kinase (PKM), catalyzes the final steps of the pathway with generation of pyruvate and ATP; two alternative splicing variants of the gene, PKM1 and PKM2, regulate the transition from glycolysis to oxidative phosphorylation [11]. PKM2 is generally expressed in cancer cells, where it promotes the Warburg effect by altering the final rate-limiting step of glycolysis. The alternative splicing of the PKM gene is regulated by miR-124, miR-137 and miR-326 in colorectal cancer (Figure 2) [11,43]. PKM2 directly interacts with the TGF (transforming growth factor)-β-induced factor homeobox 2 (TGIF2) in the nucleus of colon cancer cells, recruiting histone deacetylase 3 to the E-cadherin promoter and leading to the suppression of E-cadherin transcription and favouring epithelial-mesenchymal transition (EMT) [17]. In colorectal cancer cells, the abundantly expressed lncRNA FEZF1-AS1 binds and stabilizes PKM2, thereby activating the STAT3 signaling pathway and increasing glycolysis and dissemination potential of malignant cells (Figure 2) [12,44]. PKM2 is also regulated by miR-326 in glioblastoma cells [11,45].

In addition, other enzymes playing key roles in glucose metabolism are regulated by ncRNAs; among them, lactate dehydrogenase (LDH), which converts pyruvate to lactate. The LDHA isoform is frequently overexpressed in cancer and up-regulated by lncRNA-p21 [16]. In lung adenocarcinoma, LDHA promotes the EMT process by facilitating tumor dissemination. LDHA expression is controlled by miR-34a, miR-34c, miR-369-3p, miR-374a, and miR-4524a/b, which are commonly down-regulated in cancer tissues, such as in colorectal cancer [46]. In breast cancer, LDHA expression is instead suppressed by miR-30a-5p [47]. The LHDB isoform is controlled by miR-375, which is down-regulated in esophageal squamous cell [48] and maxillary sinus squamous cell carcinomas [49].

In lung cancer, the metastasis process has been associated with the expression of the insulin-like growth factor binding protein 4–1 (IGFBP4–1) lncRNA, through a mechanism of metabolic reprogramming; lnc-IGFBP4–1 up-regulation promotes cell proliferation and the transcription of LDHA, HK2 and PDK1, influencing energy production [50]. IGFBP4–1 is negatively associated with the expression of the gene coding for insulin-like growth factor binding protein-4 (IGFBP-4), located downstream and associated with tumor differentiation; IGFBPs mediate the effects of the insulin-like growth factors, potent mitogens that control cell proliferation in normal as well as neoplastic lung cells [50]. In lung adenocarcinoma cells, the expression of IGFBP-4 is epigenetically silenced through hypermethylation of its promoter, resulting in increased tumor proliferation and disruption of the mediated growth inhibition [51,52,53].

There is evidence that ncRNAs involved in cell metabolism take part in complex networks, not only promoting cancer development, but also playing anti-tumorigenic activities. In renal cancer cells, for example, the FoxO transcription factor has a central role in tumor suppression through the stress-induced activation of FILNC1 (FoxO-induced long non-coding RNA 1). Indeed, FILNC1 leads to the downregulation of c-Myc protein by sequestering AUF1, a c-Myc mRNA binding protein, thus inhibiting glucose metabolism and lactate production [54].

## 4. Regulation of Glutaminolysis and Mitochondrial Metabolism by lncRNAs

Genes involved in oxidative phosphorylation are significantly up-regulated in certain aggressive cancers of the breast, lung, and ovary, as well as in leukemias, lymphomas and gliomas, indicating a metabolic heterogeneity where tumor cells may use aerobic glycolysis for rapid tumor growth and mitochondrial metabolism to support malignant dissemination [55]. In addition, metabolic switch between glycolysis and oxidative phosphorylation affects the choice of the organ where tumor cells metastasize. Indeed, a study revealed that breast cancer cells usually disseminate to the liver by privileging glycolysis, while they metastasize to bone and lung when adopting oxidative phosphorylation [56]. These observation strongly suggest that a partial maintenance of mitochondrial functions might be essential for cancer cells [11].

**Figure 3:**
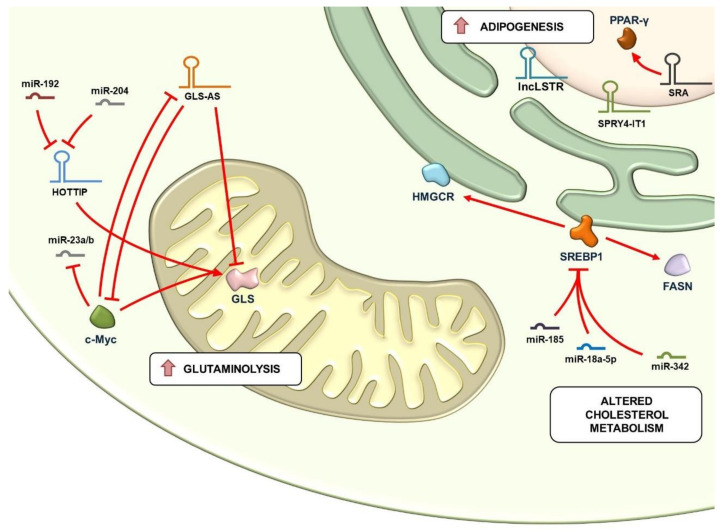
Mitochondrial and lipid metabolism are profoundly influenced by non-coding RNAs in cancer cells. The accelerated energetic demand of cancer cells is regulated by the mitochondrial enzyme glutaminase (GLS), a crucial molecular switch whose activity is finely regulated by nRNAs; miR-192 and miR-204, as an example, enhance the production of GLS via down-regulation of the HOTTIP lncRNA. c-Myc positively regulates glutamine metabolism by enhancing the expression of GLS through the suppression of miR-23A/B and/or down-regulation of GLS-AS (nuclear-located antisense lncRNA of glutaminase), which post-transcriptionally inhibits the expression of GLS. GLS-AS, in turn, can decrease c-Myc expression, thus creating a reciprocal feedback loop regulated by glutamine deprivation. Lipid metabolism is regulated by several ncRNAs; miR-185 and miR-342 regulate the activity of SREBP1 (sterol regulatory element binding protein), a master regulator of cholesterol synthesis and lipid utilization, whose target genes include, among others, FASN (fatty acid synthase) and HMGCR (3-hydroxy-3-methyl-glutaryl CoA reductase). lncRNAs implicated in lipid metabolism of cancer cells include, among others, SPRY4-IT1, LSTR, and SRA, the latter inducing adipogenesis through the activation of PPAR-gamma. Red arrows indicate a positive regulation, while truncated red arrows indicate inhibitory mechanisms.

The accelerated metabolism of cancer cells also provides increased glutamine intake and utilization (glutaminolysis), crucial for anabolic reactions of lipogenesis and nucleic acid biosynthesis supporting malignant cell proliferation [11]. Mitochondrial enzymes, such as glutaminase (GLS), play a crucial role in the metabolism of glutamine and therefore represent highly regulated molecular switches in cancer. In prostate cancer, for example, GLS expression is enhanced by the oncogenic c-Myc, which positively regulates glutamine metabolism through the suppression of miR-23A/B [11], while in HCC the HOTTIP lncRNA, deregulated by miR-192 and miR-204, enhances the production of GLS1, determining the presence of abnormal glutaminolysis [57]. The role of lncRNAs in nutrition stress and tumorigenesis has been well described in pancreatic cells, where the nuclear-located antisense lncRNA of glutaminase (GLS-AS) plays a critical role, by post-transcriptionally inhibiting the expression of GLS through its pre-mRNA targeting [58]. In pancreatic cancer cells, GLS-AS is transcriptionally down-regulated upon glutamine deprivation through nutrient stress-induced c-Myc activation, thus supporting cancer cell survival and dissemination. Low GLS-AS expression is therefore an indicator of poor clinical outcome in pancreatic cancer. GLS-AS, in turn, can decrease Myc expression, implying a reciprocal feedback loop, wherein Myc and GLS-AS regulate the expression of GLS upon nutrient stress conditions (Figure 3) [58].

## 5. Regulation of Lipid Metabolism by lncRNAs

Alterations of lipid metabolism are fairly common in cancer; adipocytes are crucial in the tumor microenvironment, representing a source of energy, hormones and signaling molecules. Reactivation of lipid biosynthesis reprogramming generates a number of biological mediators acting as second messengers that play a role in signaling pathways regulating cell growth, proliferation, differentiation and, importantly, fluidity of cytomembranes, thus participating in dissemination [12]. Recent data show that enzymes involved in lipid metabolism are regulated by ncRNAs, including miRNAs, such as miR-185 and miR-342, among whose molecular targets worth mentioning is the sterol regulatory element binding protein (SREBP1) locus, the master regulator of lipogenesis and cholesterol synthesis, and its responsive genes including fatty acid synthase (FASN) and 3-hydroxy-3-methylglutaryl CoA reductase (HMGCR) (Figure 3) [12,59]. In breast, gastric and colon carcinomas, cell–cell adhesion protein and epithelial markers are also regulated by lipid metabolism through the action of several miRNAs, targeting enzymes participating in lipid anabolic reactions, such as adenosine triphosphate citrate lyase (ACLY), acetyl-CoA carboxylase (ACC) and FASN, up-regulated in aggressive tumors and associated with metastasis. Other miRNA targets related to lipid biosynthesis include enzymes such as lipase A (LIPA), pyruvate dehydrogenase lipoamide kinase isozyme 1 (PDK1), acyl-CoA synthetase long-chain family member 1 (ACSL1) and other genes involved in lipid synthesis, including Agpat1, Mogat1, Agpat3, Agpat9, Ppap2a, and Ppap2c [12]. Several lncRNAs have been found to be associated with alterations of lipid metabolism in cancer, including SPRY4-IT1, found up-regulated in melanoma, the lncRNA SRA, that activates PPAR-gamma, inducing adipogenesis, whose up-regulation in endometrial cancer indicates a poor prognosis, or the liver-enriched lncLSTR, contributing to bile acid synthesis (Figure 3) [11]. Despite being less clear, ncRNAs also play a role in fatty acid catabolism; for example, monoacylglycerol lipase, an enzyme that hydrolyzes intracellular triglycerides, is abundantly expressed and associated with the EMT process in prostate cancer. Another hydrolytic enzyme, phospholipase D (PLD), is associated with the metastasis process [12].

## 6. lncRNAs as Biomarkers and Therapeutic Targets

There is a growing interest, nowadays, towards the identification of lncRNAs to employ as diagnostic and prognostic biomarkers in cancer; indeed, their expression is easily detectable in the saliva, serum, plasma, urine, and patient’s tissues, and can be correlated to the disease type, clinical stage and outcome. Among clinically relevant lncRNAs, the HIF-1α anti-sense lncRNA (HIFAL) plays a critical role in the transactivation of hypoxia-inducible factor-1 α (HIF-1 α), a master regulator of glucose metabolism in cancer cells; HIF-1α, in turn, induces HIFAL transcription, whose expression promotes tumor growth and is associated with an aggressive phenotype and poor outcome in breast cancer patients [60]. Another lncRNA frequently expressed in breast cancer, YIYA, interacts with cyclin-dependent kinase 6 (CDK6) and stimulates cell proliferation by increasing the glycolytic pathway; the expression of YIYA is an indicator of poor disease-free survival [61].

Interestingly, recent evidence suggests that lncRNAs might also contribute to the development of drug resistance in various cancers, either by modulating drug metabolism or by altering key signaling pathways and cellular processes, leading to drug efflux, enhanced DNA damage repair, cell cycle alterations or resistance to apoptosis [62]. In cervical cancer, for example, MALAT1 overexpression promotes the development of cisplatin resistance via the PI3K/AKT signaling pathway [63], while in hepatocellular carcinoma (HCC), the long noncoding RNA CRNDE (colorectal neoplasia differentially expressed) has been correlated with poor clinical outcome and chemoresistance by inhibiting the tumor suppressor genes CUGBP Elav-like family member 2 (CELF2) and large tumor suppressor 2 (LATS2) [64].

Due to their pleiotropic roles in cell physiology and cancer development and progression, lncRNAs have been proposed as therapeutic targets for development of anticancer drugs with minimal side-effects. A key advantage of this approach is offered by spatially and temporally restricted expression of lncRNAs: several lncRNAs are only expressed in cancer cells or in specific stages of carcinogenesis, and their inhibition can be directly adopted to modulate a particular cellular pathway, including metabolic ones. In a recent study, for example, LINC01559 and UNC5B-AS1 have been proposed as therapeutic targets as their silencing results in decreased glycolysis in pancreatic ductal adenocarcinoma (PDAC), leading to inhibition of cell proliferation [65].

In recent years, several approaches have been proposed to interfere with lncRNA expression. One of these is represented by viral vectors, namely modified adenoviruses or lentiviruses as a delivery system to introduce foreign DNA into target cells [66]. Currently, however, non-viral inhibition is preferred, due to fewer side effects and lower risk of immunogenicity; lncRNA expression can be perturbed either with small-molecule inhibitors or by employing oligonucleotide-based therapeutics (antisense oligonucleotides and RNAi mediated gene silencing). In the first case, the binding site of lncRNAs is sterically blocked by small-molecule modulators, thus preventing the lncRNA from binding to its molecular partners; alternatively, small-molecules can be used to alter the secondary structure of a target lncRNA, thus impairing the interaction with proteins or other nucleic acids and interfering with its activity [67]. This approach has been recently applied by Mercatelli et al. to interfere with the activity of the Highly Upregulated in Liver Cancer (HULC) lncRNA in Ewing sarcomas (ES), aggressive pediatric cancers of soft tissue and bone; downregulation of HULC with the small molecule YK-4-279 has been successfully employed to block an oncogenic circuit mediated by this lncRNA; from a mechanistic point of view, HULC promotes the expression of the TWIST1 oncogene in ES by sponging miR-186, which acts as a tumor suppressor in ES cells; HULC downregulation upon treatment with YK-4-279 causes the release of miR-186, thus reducing ES cell growth [68].

In addition, lncRNA transcripts can be sterically blocked by ASOs, single stranded oligonucleotides of 13–25 nt complementary to the target, usually chemically constructed to be stably introduced in cells and trigger RNAse H-mediated degradation of their target. The ASO strategy is promising for cancer therapy; for example, Gone et al. recently showed that a ASO-conjugated nanostructure targeting MALAT1 can be used to efficiently knockdown the expression of this lncRNA in the nucleus, thus reducing cell migration in lung cancer, providing a promising strategy for controlling tumor metastasis [69]. Another powerful approach to suppress the expression of a target lncRNA exploits the endogenous mechanisms for gene silencing of the RNA interference; to this end, double-stranded RNAs (dsRNAs) that are processed into short-interfering RNAs complementary to the target can be used to suppress its expression; this approach has been used from Lin and colleagues to downregulate the taurine up-regulated gene 1 (TUG1) lncRNA, an indicator of poor prognosis associated with hexokinase 2 (HK2) overexpression in HCC through the TUG1/miR-455-3p/AMPKβ2 axis, which regulates cell growth, metastasis, and glycolysis [40]. In addition, lncRNA silencing can be pursued using synthetic siRNAs mimicking the products of Dicer enzyme.

Another therapeutic strategy is offered by the possibility to increase the expression of a tumor suppressor lncRNA downregulated in cancer cells using engineered nano-vectors carrying lncRNA transcripts, in analogy with mRNA delivery. This approach has been recently proposed in HCC, where a co-delivery system based on plasmid-condensed nanocomplexes with a liver-targeting polycation gene vector has been employed to mediate the delivery of the tumor-suppressor lncRNA maternally expressed gene 3 (MEG3), resulting in the inhibition of HCC cell proliferation, migration and invasion in vitro and the inhibition of tumor growth in vivo [70].

Finally, recent evidence has shown that the CRISPR/Cas9 Genome Editing Technique can be used to target and knockout the expression of lncRNAs in cancer cells as well as in animal models. In gastric cancer, the gastric cancer metastasis associated (GMAN) lncRNA expression has been disrupted using a CRISPR/Cas9-based strategy, significantly reducing the numbers of metastases formed and improving the overall survival in mice [71]. Further studies in the field, however, are needed to design CRISPR/Cas9-based gene-editing therapies targeting lncRNAs for clinical applications.

## 7. Concluding Remarks

In conclusion, dysregulated ncRNAs participate in the metabolic reprogramming of cancer cells by regulating individual genes and modulating key molecular processes, including hypoxia-inducible pathways, the glycolytic process, oxidative phosphorylation, lipid anabolic and catabolic reactions, amino acid metabolism and signal transduction pathways. The complex network of interactions established by cancer cells with the contribution of metabolism-regulated ncRNAs affect cancer growth, differentiative state, metastasis potential and therapy. For their pleiotropic function, which can be of structural and/or regulatory nature, the lncRNA class generated a great interest in the scientific community; indeed, due to their expression, frequently dysregulated in cancer, lncRNAs are currently guarded as promising diagnostic and prognostic biomarkers; however, how the lncRNAs fine-tune the onset of cancer remains to be investigated. In addition, because of their tissue specificity, several studies highlighted their potential as new targets for personalized gene-editing therapies with innovative technologies and minimal side-effects, such as the ASO/siRNA and the CRISPR/Cas9 approach. Intriguing questions on lncRNA biology still require further investigation, including the dynamics of interactions of lncRNAs with proteins and other molecules involved in metabolism circuits, their generally low-sequence conservation and structural features related to their function, their subcellular localization, accumulation in subcellular compartments and circulating exosomes etc. Progress in this field will be of great relevance, offering a better understanding on how metabolism-associated lncRNAs regulate cancer cell survival and disease progression. Thus, due to their wide range of interactions and connections with key cellular pathways frequently dysregulated in cancer, metabolism-associated lncRNAs may provide a novel approach for the early diagnosis and personalized therapy of many kinds of malignancies.

## Abbreviations

ACC	Acetyl-CoA carboxylase
ACLY	adenosine triphosphate citrate lyase
ACSL1	acyl-CoA synthetase long-chain family member 1
AMPK	AMP-activated protein kinase
AMPKβ2	adenosine monophosphate-activated protein kinase subunit β2
AMPKβ2	adenosine monophosphate-activated protein kinase subunit β2
CDK6	cyclin-dependent kinase 6
CELF2	CUGBP Elav-like family member 2
CRNDE	Colorectal Neoplasia Differentially Expressed
dsRNAs	double-strand RNAs
EMT	epithelial-mesenchymal transition
ES	Ewing sarcoma
FASN	Fatty acid synthase
FILNC1	FoxO-induced long non-coding RNA 1
GLS	Glutaminase
GLS-AS	nuclear-located antisense lncRNA of glutaminase
GLUTs	Glucose transporters
GMAN	Gastric cancer metastasis associated
HCC	Hepatocellular carcinoma
HIF-1 α	Hypoxia-inducible factor-1 α
HIF-1α	Hypoxia-inducible factor 1-alpha
HIFAL	HIF-1α anti-sense lncRNA
HK2	Hexokinase 2
HKs	Hexokinases
HMGCR	3-hydroxy-3-methyl-glutaryl CoA reductase)
HOTAIR	HOX Transcript Antisense Intergenic RNA
HULC	Highly Upregulated in Liver Cancer lncRNA
IGF	insulin-like growth factor
IGFBP4–1	insulin-like growth factor binding protein 4–1
LATS2	large tumor suppressor 2
LDH	lactate dehydrogenase
lincRNAs	long intergenic non-coding RNA
LIPA	lipase A
LKB1	liver kinase B1
lncRNAs	long non-coding RNA
MALAT1	Metastasis Associated Lung Adenocarcinoma Transcript 1
MEG3	maternally expressed gene 3
MMP-2	matrix metalloproteinase-2
NATs	natural antisense transcripts
NBR2	neighbor of BRCA1 gene 2
NRCP	ceruloplasmin lncRNA
PDAC	pancreatic ductal adenocarcinoma
PDK1	pyruvate dehydrogenase lipoamide kinase isozyme 1
PKM	Pyruvate kinase
PLD	phospholipase D
sncRNAs	small non-coding RNAs
SREBP1	sterol regulatory element binding protein
TGIF2	transforming growth factor-β-induced factor homeobox 2
TRINGS	Tp53-regulated inhibitor of necrosis under glucose starvation
T-UCR	transcribed ultraconserved region
TUG1	taurine up-regulated gene 1
